# The Influence of Reactive Oxygen Species in the Immune System and Pathogenesis of Multiple Sclerosis

**DOI:** 10.1155/2020/5793817

**Published:** 2020-06-25

**Authors:** Mohammad javad Tavassolifar, Mohammad Vodjgani, Zahra Salehi, Maryam Izad

**Affiliations:** ^1^Immunology Department, School of Medicine, Tehran University of Medical Sciences, Tehran, Iran; ^2^MS Research Center, Neuroscience Institute, Tehran University of Medical Sciences, Tehran, Iran

## Abstract

Multiple roles have been indicated for reactive oxygen species (ROS) in the immune system in recent years. ROS have been extensively studied due to their ability to damage DNA and other subcellular structures. Noticeably, they have been identified as a pivotal second messenger for T-cell receptor signaling and T-cell activation and participate in antigen cross-presentation and chemotaxis. As an agent with direct toxic effects on cells, ROS lead to the initiation of the autoimmune response. Moreover, ROS levels are regulated by antioxidant systems, which include enzymatic and nonenzymatic antioxidants. Enzymatic antioxidants include superoxide dismutase, catalase, glutathione peroxidase, and glutathione reductase. Nonenzymatic antioxidants contain vitamins C, A, and E, glutathione, and thioredoxin. Particularly, cellular antioxidant systems have important functions in maintaining the redox system homeostasis. This review will discuss the significant roles of ROS generation and antioxidant systems under normal conditions, in the immune system, and pathogenesis of multiple sclerosis.

## 1. Introduction

The oxidation reaction is the reaction in which the electrons passed from one substance (reactant) to another one. This reaction results in the production of ROS [1]. Fenton explained reactive oxygen species (ROS) for the first time in 1894, which are also known as free radicals [2]. The most important type of radicals produced in living systems is ROS [3]. Superoxide anion (O_2_^−^), hydrogen peroxide (H_2_O_2_), and hydroxyl radical (OH^−^) are three main types of ROS [1]. Physiological concentrations of ROS as second messengers are required to maintain the cellular state including cell signaling, differentiation, proliferation, and growth, apoptosis, and cytoskeletal regulation. High local concentrations of ROS are produced by immune cells to kill pathogens [4], while the much higher concentration of ROS can damage cellular components such as proteins, lipids, and nucleic acids [5]. In this review, we will discuss how ROS and antioxidants are produced in mammalian cells and the effects of ROS on immune cell function and in the pathogenesis of multiple sclerosis.

## 2. Reactive Oxygen Species Generation

Reactive oxygen species (ROS) are produced by a diversity of extracellular and intracellular agents [6]. Exogenous stimuli such as UV radiation, air pollution, smoking, and environmental chemicals cause ROS generation [7]. Electron leak from the mitochondrial respiratory chain and NADPH oxidases are two major intracellular sources of ROS.

### 2.1. Superoxide and Hydrogen Peroxide Generation in the Mitochondria

When electrons escape the Electron Transfer Chain (ETC) in the mitochondria, ROS are produced [8]. Electrons are transferred to complex I by nicotinamide adenine dinucleotide (NADH) and then transferred to complex II through dihydroflavin adenine dinucleotide (FADH2), both of which are located within the mitochondrial membrane. Afterward, the electrons are transferred through ubiquinone and cytochrome C to complex III and complex IV, respectively. Finally, H_2_O is produced by electrons transferred to O_2_. Although this reaction is highly conserved, sometimes electrons can leak by complexes involved in the electron transport chain to partially reduce O_2_, causing the production of superoxide radicals [9, 10]. Therefore, the first free radical produced through the electron transport chain in mitochondria is superoxide anion (O_2_^−^) [11]. Superoxide anion is the precursor of the other two ROS, H_2_O_2_ and OH^−^. Superoxide anion is spontaneously converted into hydrogen peroxide when an electron and two protons are added to O_2_^−^. Furthermore, superoxide dismutase can convert superoxide to H_2_O_2_ [12]. Then, H_2_O_2_ breaks down into hydroxyl radical (OH) and a hydroxide ion. Hydroxyl radicals can be also produced from the interaction between O_2_^−^ radical and hydrogen peroxide in the Haber–Weiss reaction [1, 13]. Hydroxyl radical, the most powerful and harmful radical among the ROS, is able to interact with all biological macromolecules such as lipids, proteins, nucleic acids, and carbohydrates [14].

### 2.2. Production of ROS by NADPH Oxidase

In addition to mitochondria, intracellular ROS are generated by nicotinamide adenine dinucleotide phosphate oxidase (NOX) protein family. Nicotinamide adenine dinucleotide oxidase is a flavocytochrome that was initially found in phagocytes. It is involved in destroying pathogens by releasing superoxide or hydrogen peroxide in the phagocytic compartment [15].

The NOX family consists of seven members (NOX1-5 and DUOX1-2), which are divided into three groups based on the presence of domains other than the gp91phox (NADPH oxidase-2 (NOX2)) domain [16]. The first group including NOX1, NOX3, and NOX4, have a similarity in size and structure with gp91phox [17]. NOX5, as the second group, has an amino-terminal calmodulin-like domain that contains four binding sites for calcium. The NOX5 is expressed upon activation of the calcium ionophore ionomycin. The third group contains Duox1 and Duox2 which have a peroxidase-homology domain and uses ROS produced by the catalytic core to produce more potent oxidizing species, which leads to oxidation of extracellular cells [18].

NADPH oxidase consists of four cytosolic subunits p47phox, p40phox, p67phox, and a small G-protein Rac (p21 Rac), as well as gp91phox and p22phox, both known as functional transmembrane heterodimers. NADPH oxidase is activated by the assembly of these regulatory subunits with gp91phox [18, 19].

In the resting cells, Rac2 via interaction with RhoGDI remains inactive in the guanosine diphosphate (GDP) bound state, while gp91phox and p22phox reside in intracellular vesicles. After starting phagocytosis, GDP-Rac2 by the activity of a Rac guanine nucleotide exchange factor is converted to GTP-Rac2. Then, Rac2 is transferred to the plasma or phagosome membrane. In addition, gp91phox and p22phox are transferred from the vesicle to the membrane [20, 21]. At the same time, p47phox is phosphorylated and goes under conformational change, which currently places two regions of SRC homology 3 in conjunction with proline-rich motifs in p22phox [19]. Additionally, via Phox homology domains, p47phox binds to phosphatidylinositol 3-phosphate (PI3P) and PIP2 [3, 4] stabilizing the p47phox position in cytochrome b558. When p47phox, p67phox, and p40phox are trimerized in the cytosol, the other two regulatory subunits are bound by p47phox [22].

After assembly and activation, NADPH oxidase generates superoxide by converting NADPH to NADP^+^, leading to the release of two electrons and one H^+^; then cytochrome b558 transfers two electrons to the lumen of the phagosome which in turn reacts with two molecules of oxygen to form two superoxide ions [19]. Isoforms of NOX exist in the plasma membrane, as well as intracellular membranes of the nucleus, mitochondria, and endoplasmic reticulum. Depending on its location, O_2_^−^ can be released into intracellular or extracellular space [15].

## 3. Defense Mechanisms against Reactive Oxygen Species

All cells have an intrinsic mechanism that neutralizes excess ROS and minimizes the adverse effects caused by them, the so-called antioxidant system [23]. Antioxidants are divided into small molecules and enzymatic antioxidants. Small molecules also known as antioxidants contain vitamins C, A, and E, uric acid, antioxidant minerals (copper, ferritin, zinc, manganese, and selenium), L-*γ*-glutamyl-L-cysteinylglycine (GSH), pycnogenol, and thioredoxin, which prevent cellular damage [24]. Enzymatic antioxidants include superoxide dismutase (SOD), catalase (CAT), glutathione peroxidase (GPx), glutathione reductase (GR), and thioredoxin reductase [25]. The expression of antioxidant enzymes is controlled by the transcription factor nuclear factor-E2-related factor (Nrf2) [26].

### 3.1. Superoxide Dismutases (SODs), Catalase, and Peroxiredoxins

**Figure 1:**
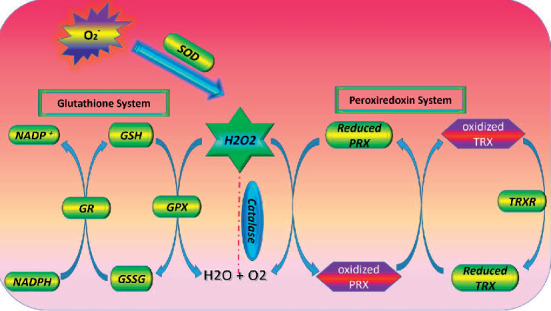
Antioxidant enzymes. The superoxide radical anion can be transformed either to H_2_O_2_ SOD. The hydrogen peroxide can be converted to H_2_O and O_2_ by CAT and/or GPX/PRX. H_2_O_2_, hydrogen peroxide; SOD, superoxide dismutase; CAT, catalase; GPX, glutathione peroxidase; PRX, peroxiredoxin enzymes.

The first line of the oxidative stress defense system against ROS is the superoxide dismutases (SODs) and catalase. Superoxide dismutases have four isoforms including Mn-SOD, Cu, Zn-SOD, Ni-SOD, and extracellular SOD. These enzymes convert superoxide radicals to oxygen and hydrogen peroxide [27]. Catalase can convert H_2_O_2_ to form water and molecular oxygen. Excessive expression of catalase in cytosol or mitochondria has been shown to protect cells from oxidative damage [28]. Another H_2_O_2_ scavenger is peroxiredoxins (PRX) (six isoforms of PRXs have been known). Through the oxidation of its own cysteine residues, PRX causes a reduction of H_2_O_2_ to H_2_O which gets inactivated. Hence, the cysteine residues of thioredoxin (TRX) are oxidized as the inactivated PRX is reactivated [29] (Figure 1).

### 3.2. Glutathione

One of the most important antioxidant systems in the cell is the glutathione system. The glutathione system contains glutathione peroxidase (GPX), glutathione reductase (GR), and glutathione. Glutathione consists of L-*γ*-glutamyl-L-cysteine-y-L-glycine (g-Glu-Cys-Gly) which has a nonprotein thiol group of a cysteine residue [30]. Glutathione exists in two forms including a reduced form (GSH) and an oxidized form, called glutathione-disulfide (GSSG) [31].

Indeed, GSH acts as an electron donor that causes the reduction of any disulfide bonds formed within cysteines of cytoplasmic proteins [31]. Therefore, GSH can prevent interactions between oxidizing species and cellular components, especially nucleic acids and proteins. Additionally, GSH acting as cosubstrate to the enzyme glutathione peroxidase (GPX) and glutathione reductase (GR) leads to a reduction of peroxides. So far, eight isoforms of GPXs have been recognized in the cytosol, mitochondria, endoplasmic reticulum, peroxisomes, and extracellular space [32, 33]. Similar to PRX and TRX, GPX induces the conversion of hydrogen peroxide into two molecules of water through the oxidation of GSH. Then, glutathione reductase (GR) uses NADPH to convert oxidized glutathione (GSSG) into GSH, a process that leads to regenerating the pool of glutathione in cells [33] (Figure 1).

### 3.3. Nrf2-Keap1-ARE Pathway

Antioxidant systems are regulated at both mRNA expression and protein enzymatic activity level; the former is regulated by Keap1-Nrf2-ARE [34], leading to maintaining the redox balance in the cells. The Nrf2-Keap1-Cul3 trimeric complex is divided into three main cellular components: Kelch-like ECH-associated protein 1 (Keap1), which is a known inhibitor of Nrf2 (INrf2), nuclear factor erythroid 2-related factor 2 (Nrf2), and antioxidant response element (ARE), which is known as a common promoter element of genes. Under homeostatic conditions, Keap1 acts as an inhibitor, which interacts with Nrf2 in the cytoplasm, preventing Nrf2 translocation to the nucleus. Afterward, Nrf2-Keap1 binding to the E3 ubiquitin ligase Cullin 3 (Cul3) causes ubiquitination of Nrf2, eventually degraded by the 26S proteasome [35, 36]. When oxidative stress occurs in cells, ROS can induce the breakdown of the Nrf2 and Keap1, through the oxidation of cysteine residues (Cys273, Cys288, and Cys151) and the activation of kinases, like protein kinase C (PKC), MAPK, phosphatidylinositide 3-kinases (PI3Ks), and protein kinase-like endoplasmic reticulum kinase (PERK), causing phosphorylation of Nrf2 [34]. Subsequently, Nrf2 is stabilized and transferred to the nucleus where it dimerizes with small Maf proteins (Maf-F, Maf-G, and Maf-K). Then the heterodimers interact with ARE, resulting in the activation of antioxidant response genes [37]. There are reportedly almost 200 induced genes involved in detoxification and antioxidant defense by Nrf2, including SODs, glutathione synthetase (GSS), GR, GPXs, thioredoxin (TRX), thioredoxin reductase (TRR), peroxiredoxin (PRX), and catalase [38].

The disturbance in the balance between oxidants and antioxidants is called “oxidative stress” (OS) which causes impairment in redox signaling and molecular damage [39].

## 4. Roles of Reactive Oxygen Species in the Immune System

So far, the reports show that redox states have important roles in immunity and T-cell function. Engagement of T-cell receptor (TCR) with APC results in the production of intracellular ROS. Two variables determine the effects of ROS on adaptive immune responses, the lymphocyte subset, and intracellular or extracellular ROS [19, 40]. Moreover, the ROS levels affect adaptive immune responses, such that a minimal increase in ROS level in the immune system may raise normal immune function, whereas high levels of ROS result in raised levels of proinflammatory cytokines through the loss of Nrf2 [41]. Moderate levels of ROS can act as biochemical mediators in immunity involved in several cellular functions, primarily bacterial defense, cell proliferation, aggregation, chemotaxis, Ag processing, and signaling pathways [42].

### 4.1. Microbicidal Activity

One of the earliest and most powerful defensive mechanisms against invading microorganisms in phagocytes is NOX-derived-superoxide in which NADPH oxidase is activated after the ingestion of microorganisms into the phagosome [43]. Superoxide anion and other products such as H_2_O_2_ and HOCl can kill microorganisms via destroying iron-sulfur centers such as those in the respiratory chains of microorganisms. Furthermore, O_2_^−^ indirectly kills microorganisms via proteolytic digestion of microorganisms. Indeed, O_2_^−^ leads to depolarization of the phagosome membrane and a compensating influx of H^+^ and K^+^ into the phagolysosome, which removes proteases from the anionic proteoglycan matrix by raising ionic strength [44, 45].

### 4.2. Effects of ROS on Antigen Cross-Presentation

Antigen cross-presentation, a process that can present extracellular antigens with MHCI molecules to CD8^+^ T cells, leads to the initiation of CD8^+^ T-cell responses [46]. When exogenous antigens are taken up via phagocytosis and partially degraded, they can be exported into the cytosol, where they are degraded by the proteasome and then transferred to the ER for loading on MHCI molecules [19, 47]. Studies have demonstrated that one of the ROS roles in the immunity system is the regulation of antigen cross-presentation via the effect on the phagosome pH [48]. ROS production in DC phagosomes increases pH level not only through consuming protons (H^+^) but also through oxidation of two cysteine residues within the nucleotide-binding subunits of the enzyme which results in the inactivation of V-ATPase. Consequently, Ag is protected against endosomal degradation and transported to the cytosol [49]. Studies have shown that DCs isolated from patients with the chronic granulomatous disease (CGD) or treated with NOX2 inhibitor demonstrate impaired antigen cross-presentation [50].

### 4.3. Chemotaxis

Chemotaxis is a biological process in which cells migrate in response to chemoattractant gradient, which can regulate the migration of leukocytes such as neutrophils, monocytes, and effector T cells, and modulate inflammatory reactions. Some studies have shown that ROS play an important role in the regulation of leukocyte chemotaxis to the sites of injury or infection [51]. Studies have shown that neutrophils isolated from CGD patients or treated with NOX2 NADPH oxidase inhibitor, diphenyleneiodonium chloride (DPI), or siRNA have silenced gp91phox or p22phox causing faulty migration due to the inhibition of ROS production [52]. When the herpes virus entry mediator (HVEM) interacts with LIGHT, a known member of the tumor necrosis factor (TNF) family, it causes increased infiltration of both macrophages and neutrophils and expression of chemokine receptors like CCR1 and CCR2, through increasing ROS production [19]. Besides, the migration process in macrophages is regulated by Nox2-dependent ROS [53]. For instance, in angiotensin II-induced hypertension model, macrophage infiltration into vessels is reduced through the inhibition of Nox2 activity, while lacking Nox2 oxidase activity reduces macrophage infiltration into atherosclerotic lesions [54].

### 4.4. Immune Modulatory

In addition to its antimicrobial activity, ROS is also associated with the progression of inflammatory diseases via increased biological structures and proinflammatory cytokines; however, recent observations illustrated that ROS can act as a regulatory agent in the immune system [19]. Treg function and ROS level have a close relationship with each other. ROS levels divided the mildly high tolerable levels or the intolerably higher range, in the latter of which, ROS causes tissue damage and inflammatory reactions. However, at tolerable levels, ROS can act as an anti-inflammatory agent in the immune system. Efimova et al. showed that hypofunctional Tregs are associated with lower levels of ROS [55]. In the same vein, Kim et al. demonstrated the Treg function elevated by increased levels of ROS. Superoxide radicals can act as a cofactor for IDO. Therefore, the enzyme activity of IDO elevated by increased levels of ROS [56].

### 4.5. Role of ROS as Second Messengers in Cellular Signaling

Intracellular ROS, also known as second messengers, are involved in some cellular functions such as cellular differentiation, tissue regeneration, and aging prevention [57]. The two factors that determine whether the redox systems act as redox signaling or oxidative stress are the type of ROS and its local concentration. Studies have demonstrated that the effects of the accumulation of superoxide on redox signaling are lower than the oxidative stress [58]. One important feature that enables H_2_O_2_ to act as an intracellular signaling molecule is its potential for quick diffusion through membranes [59]. Hence, H_2_O_2_ signal transduction occurred through selectively oxidizing cysteine residues within proteins. Redox signaling by H_2_O_2_ is also associated with the source of generated H_2_O_2_. For instance, when H_2_O_2_ is produced by NADPH oxidases in the plasma membrane, it targets the plasma membrane proteins [60]. ROS can regulate signaling not only through oxidizing cysteine residues but also by the modulation of Ca^2+^ signaling. ROS can also release Ca^2+^ by activating the inositol triphosphate (IP3) receptor and oxidation of the ryanodine receptor, raising the intracellular concentration of Ca^2+^ in immune cells, and regulating differentiation, proliferation, and activation [19]. H_2_O_2_ can convert cysteine residues to cysteine sulfenic acid (Cys-SOH) by oxidation, leading to the formation of disulfide bonds, which in turn alter the protein function. While higher levels of H_2_O_2_ transform oxidized thiolate anions to sulfinic (SO_2_H) or sulfonic (SO_3_H) species, this species results in permanent protein damage [58].

Activation of T cells depends on three types of signals: recognizing antigen via TCR-MHC complexes; the first signal, interactions of costimulatory molecules; the second signal, ROS and proinflammatory cytokines; the third signal required for efficient activation of T cells [61]. The stimulation of TCR produces two species of oxidants, which tend to regulate signal transduction through different pathways. For instance, hydrogen peroxide regulates the proliferative signal through ERK activation, while O_2_^−^ enhances a prodeath signal in mature T cells via inducing FasL expression [62]. Therefore, ROS are known as second messengers which enhance TCR signaling through oxidation of calcium and potassium channels, integrins, CD2, the TCR/CD3 complex, and ZAP70. Further, ROS causes an activation of the ERK and mitogen-activated protein kinase (MAPK) family member [48].

#### 4.5.1. MAPK/ERK Pathway

The MAPK/ERK pathway, known as the Ras-Raf-MEK-ERK pathway, is necessary to transduction of biological signals from the cell membrane to the nucleus [63]. The MAPK/ERK pathway is activated by several extracellular signals such as growth factors, cytokines, and T-cell receptor antigens [64]. Ligand binding to cell surface receptors induces activation of the small GTPase Ras, followed by MAP kinase kinases Raf activation by phosphorylation [65]. Afterward, activated Raf leads to activation of MAP kinase kinases MEK1/MEK2, and then MAP kinases ERK1 and ERK2 are phosphorylated and activated by MEK1/MEK2. Finally, ERK1/2 serves in the phosphorylation of cytosolic substrates such as RSK [65, 66]. ERK1/2 can be transferred to the nucleus and activate transcription factors such as Elk-1 [8]. ERK1/2 phosphorylation is regulated by two signaling pathways in T cells [67]. The first signaling pathway mediates MAPKKK, MAPKK, and MAPK by phosphorylation in the classical MAPK pathway. ERK1/2 phosphatases such as He-PTP can regulate the MAPK pathway in the other pathway signaling. Oxidative modification of intracellular kinases like ASK-1 by ROS leads to the activation of MAPK signaling cascade [68, 69]. Increased upstream signaling pathways (e.g., MEK1/2 activation) by H_2_O_2_ result in enhanced phosphorylation levels of ERK1/2 and MEK1/2, which in turn causes Lck shift from 56 to 60 kDa. One of the negatively regulated TCR signaling enzymes is protein tyrosine phosphatases (PTPs) which have cysteine residue in the active site and sensitive to oxidation. H_2_O_2_ acts as a general inhibitor of PTPs, converting Cys residue active site to sulfenic acid (Cys-SOH). Therefore, H_2_O_2_ activates downstream signals such as MAPK by activating phosphorylation cascades through the inhibition of protein tyrosine phosphatases activity [67, 70, 71].

**Figure 2:**
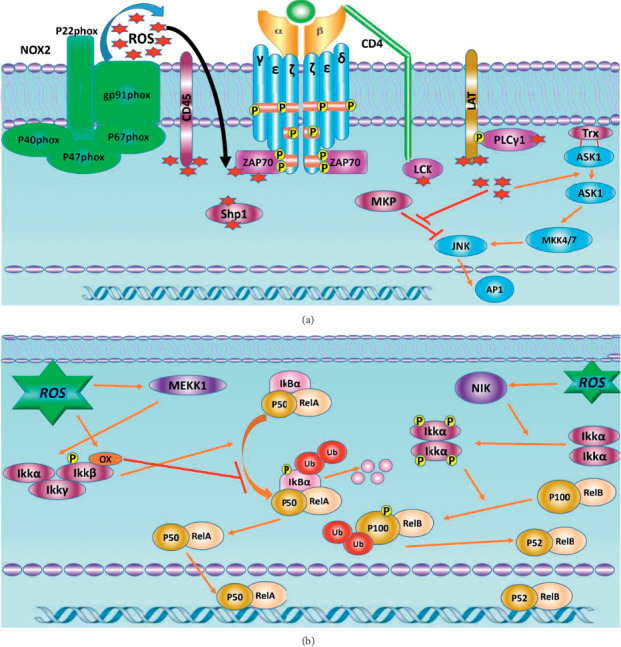
ROS effects on TCR signaling. TCR engagement leads to ROS production (red stars) from NOX2 at the plasma membrane. (a) Possible targets of ROS including LAT, ZAP70, SHP-1, Lck, and CD45. (b) Crosstalk of ROS with NF-*κ*B signaling pathways. TCR, T-cell receptor; NOX2, NADPH oxidase 2; ROS, Reactive oxygen species; LAT, linker for activation of T cell; ZAP70, Zeta Chain Of T-Cell Receptor Associated Protein Kinase 70; SHP-1, Src homology 2 domain-containing protein tyrosine phosphatase 1; Lck, lymphocyte-specific protein tyrosine kinase; MEKK1, mitogen-activated protein kinase kinase kinase 1; NIK, NF-*κ*B inducing kinase.

One of the important MAPK signaling pathways is the c-Jun N-terminal kinases (JNKs), also called stress-activated protein kinases (SAPKs), which are activated via inflammatory cytokines and oxidative stress [72]. The JNKs are involved in some cellular processes, including cell differentiation and cellular apoptosis pathways [73]. Like the ERK pathway, the JNK signaling pathways including MAPKKK (which contains MEKK1, MEKK2, MEKK3, and MEKK4, MLK, and ASK1) and MAPKK consist of MKK4, MKK3, MKK6, and MKK7. Phosphorylation of JNK on critical threonine and tyrosine residues by activated MAPKKK and MAPKK resulting in a transfer of JNK into the nucleus is able to activate transcription factors such as c-Jun, ATF, and Elk1 [74, 75]. Under physiological conditions, thioredoxin (Trx) is an antioxidant protein, which binds to ASK1, and causes the inhibition of ASK1 kinase activity; however, the oxidative stress leads to ASK1 separating from Trx by changing the conformation of Trx; then, ASK1 could be oligomerized and autophosphorylated [64, 76]. Glutathione S-transferase pi (GSTp) is a member of the glutathione S-transferase family of enzymes, acting as detoxification of electrophilic metabolites, which can inhibit JNK signaling pathway via the interaction with JNK, but ROS induces activation of JNK through separating JNK from glutathione S-transferase pi (GSTp) [77] (Figure 2(a)).

#### 4.5.2. NF-*κ*B Pathway

NF-*κ*B proteins are important transcription factors acting in inflammation and immunity, which can be regulated by ROS [78]. Phosphorylation of Tyr42 or other tyrosine residues of I*κ*B*α* via H_2_O_2_ leads to degradation of I*κ*B*α* and ultimately activation of the NF-*κ*B pathway [79]. It has been shown that overexpression of antioxidant proteins such as TRX1 leads to reduced NF-*κ*B activation by inhibiting I*κ*B degradation [78]. The MEKK1, also known as kinases upstream of IKK and redox-sensitive kinase, could be inhibited by glutathionylation at C1238 through ROS [80]. H_2_O_2_ can transfer NF-*κ*B into the nucleus through phosphorylation serine of the p65 subunit of NF-*κ*B. An NF-*κ*B-inducing kinase (NIK), also known as an upstream kinase in the NF-*κ*B pathway, is activated through increasing phosphorylation by ROS [81] (Figure 2(b)).

#### 4.5.3. Phospholipase C-*γ*1 and Calcium

Through producing second messengers such as diacylglycerol and inositol 1,4,5-trisphosphate, phospholipase C-*γ*1 (PLC-*γ*1) induces the activation of protein kinase C (PKC) and increases intracellular calcium level, which in turn regulates cell proliferation and differentiation. Phosphorylation tyrosine of PLC-*γ*1 by tyrosine kinases such as Src family kinases activates PLC-*γ*1. The ROS could activate Src family tyrosine kinases, which then phosphorylate PLC-*γ*1 and PLC-*γ*2. PI3–K/Akt involved in signaling events is stimulated by H_2_O_2_, which activates PLC-*γ*1 [82, 83].

Calcium channels like RyR, IP3R, SERCA, PMCA, and NCX are involved in the regulation of many signaling pathways. RyR/IP3R plays an important role in channel gating and assembly by posttranslational modifications of Cys residues through redox modifications [84]. Therefore, ROS enhances channel activity by thiol oxidation of RyR/IP3R. ROS inhibits SERCA activity by oxidation of Cys residues of SERCA pumps, thus reducing Ca^2+^ influx from the cytoplasm to ER [85].

### 4.6. Relationship between ROS Signaling and the Immune System

The TCR is cross-linked when a naive T cell binds to its cognate antigen, causing the activation of a variety of signaling cascades and transcription factors that induce rapid T-cell proliferation in response to infection. The MAPK family is an important class of signaling proteins appropriate for this process. The MAPK pathway is known to be triggered early after the TCR binds to the antigen, which results in the activation of several essential cell transcription factors for inducing T-cell growth and survival [86]. Several studies have already shown that ROS signaling in activated T cells may be one mechanism by which the MAPK pathway is regulated [87]. Mitochondrial ROS (MtROS) function on the proinflammatory signaling pathways trigger the production of cytokines. The key pathways causing cytokine production are the MAPK pathways ERK1/2, JNK1/2, and p38 and the pathways leading to NF-*κ*B activation [88]. The MtROS can also induce proinflammatory cytokine production in an inflammasome-independent manner, likely by inactivating MAPK phosphatase which adversely regulates the transcription of the proinflammatory cytokine gene [89]. The MtROS contributes to the production of NF-AT and IL-2 in T cell as a result of the ROS-mediated modification of kinases' redox status, which modulates the NF-AT and IL-2 pathways to promote proliferation after stimulation [90]. Increased generation of ROS as a result of inflammatory signaling will mediate canonical activation of NF-*κ*B and downstream induction of inflammatory genes, activation of inflammasomes, and secretion of cytokines [91]. NOX-2-induced ROS enhances interferon- (IFN-) *γ* synthesis by increasing the rates of JNK and NF-*κ*B phosphorylation, STAT-1, and T-bet transcription factors, and IL-2, IL-4, TNF-*α*, and GM-CSF cytokine secretion. In addition, the NOX-2-derived ROS reduces STAT3 phosphorylation and IL-10, TGF-*β*, and IL-17 production [92, 93].

### 4.7. Effects of ROS on T-Cell Differentiation

Many researchers have identified ROS as an agent capable of modulating the differentiation of various T-cell subsets through the regulation of GATA-binding protein 3, signal transducer and activator of transcription, and T-box transcription factor [86]. The evidence has shown the expression of transcription factors STAT1, STAT4, and T-bet and production of cytokines, such as IL-2, IL-4, IFN-*γ*, and TNF-*α*, to be diminished in T cells of NOX-deficient animals. Moreover, phosphorylation of STAT3 and the production of IL-10, TGF-*β*, and IL-17 have been shown to be increased in p47phox deficient T cells [94]. In NOX-deficient mice, T cells demonstrate lessened IL-4 and augmented IL-17 production, displaying Th17 phenotype [92]. Additionally, in mouse models of IEX-1 gene deficiency as well as those treated with mitochondria ROS inhibitors like N-acetylcysteine and mitoquinone, differentiation to Th17 phenotype is reduced [95, 96]. Inhibition of ROS sources via the utilization of antioxidants leads to increased IFN-*γ* production in T cells, promoting Th1-mediated immune responses. Hence, increased levels of ROS triggered to promote both IL-4 and IL-2 production in T cells lead to T-cell differentiation towards the Th2 phenotype [86, 97]. Several studies have mentioned the important role of Nrf2 in the activation and differentiation of T cells. Findings indicate the decrease of expression levels of activation markers such as CD25 and CD69, as well as decreased production of IL-2, in T cells treated with Nrf2 activators [98]. The mouse model with Keap1 deficiency shows systemic activation of Nrf2 and, similar to cells treated with Nrf2 activators, shows decreased IFN-*γ* and increased IL-4, IL-5, and IL-13 production. They also prevent T-bet binding to DNA while enhancing GATA-3 ability to bind to DNA. Therefore, these studies reveal that the differentiation of inflammatory Th cell subsets can be inhibited via Nrf2 [99, 100].

### 4.8. ROS and T-Cell Death

The elimination of activated lymphocytes during the terminal phase of the immune response occurs in cytokine or the activation-induced cell death (AICD). However, a small portion of the expanded T cells remain which provided immune protection when encountering the same antigen again [101]. The latter mechanism is necessary for preserving the homeostasis of T cells and is mainly regulated by the death receptor Fas (CD95) [102]. The resting T cells expressed a low level of CD95; however, the activated T cells increased the level of CD95 L through the induction of NF-AT or NF-*κ*B [103]. Endogenous superoxide, which can induce the expression of CD95 L in T cells via activating NF-*κ*B, causes AICD [104]. In T cells, the survival of naive cells depends primarily on high Bcl-2 expression, while in antigen-specific T cells, the amount of Bcl-2 decreases significantly at the immune response [89]. Therefore, Bcl-2 expression is downregulated via ROS that causes intrinsic apoptosis [105]. ROS scavenger such as GSH can decrease ROS-induced apoptosis of naive and memory T cells [106]. Several studies have also shown an association between the progression of apoptosis and intracellular GSH depletion [92, 106].

### 4.9. Mitochondrial Hyperpolarization and T Cells

Mitochondrial metabolic activity plays a crucial role in regulating the activation, proliferation, and programmed death of T cells [107]. Producing reactive oxygen intermediates (ROI) and ATP synthesis, which is strictly controlled by the mitochondrial transmembrane potential (ΔΨm), induces T-cell activation and death pathway selection [108]. T-cell mitochondrial hyperpolarization (MHP) has been correlated with increased levels of cellular ROS [109]. T-cell activation via TCR/CD28 costimulation leads to MHP, which is correlated with temporary inhibition of F0F1-ATPase, increased ROI generation, ATP depletion, and necrosis sensitivity. Therefore, ΔΨm elevation seems to be a critical checkpoint for T-cell fate [110]. The studies have shown that the production of MHP is an early event preceding phosphatidylserine (PS) externalization, activation of caspases, and destruction of ΔΨm in H_2_O_2_ [29] and Fas-induced apoptosis in Jurkat human leukemia T cells and normal human peripheral blood lymphocytes (PBLs) [111]. H_2_O_2_ via enhancing ΔΨm and production of ROI causes induction of apoptosis of PBLs in healthy subjects, which need mitochondrial conversion into an ROI. For instance, OH occurred through the Fenton reaction [112]. Furthermore, elevated baseline ΔΨm and levels of ROI could have major roles in altered activation and spontaneous death of lupus T cells [110]. Further, CD4^+^ T cells with MHP secrete higher levels of IFN-*γ*, which propose a significant role of MHP in CD4^+^ T-cell differentiation into TH1 lineage3 [113].

## 5. Role of ROS in the Mechanisms of MS Pathogenesis

Apart from the physiological conditions, ROS levels can affect an adaptive immune system, as well as serving as a risk factor in the development and progression of autoimmune disorders [23]. Moreover, the antioxidant system, mainly GSH, has been impaired in chronic degenerative diseases, particularly inflammatory immune-mediated ones [31]. Hence, through the production of novel autoantigens, oxidative stress leads to the autoimmune response [1]. Cumulating evidence has implicated ROS in neuroinflammatory disorders such as multiple sclerosis (MS), causing cellular injury via overwhelming the antioxidant capacity [114].

Multiple sclerosis is an autoimmune disease of the central nervous system (CNS), characterized by demyelination and axonal injury. Several studies have indicated the onset of MS between the ages of 20 and 40, but some reports have revealed that MS may occur in young children and elderly adults. The cause of MS has not clearly elucidated. It is described as a multifactorial disease. Several reports have revealed genetic and environmental factors as well as inflammation and oxidative stress as key players in the pathogenesis of MS [115, 116]. The initial events in the formation of MS include autoreactive CD4^+^ T cells recognizing myelin-like peptides presented by antigen-presenting cells in the periphery and subsequently infiltrating into the CNS through BBB and then reactivating in the CNS by the respective antigen, leading to loss of myelin in the brain and spinal cord. Numerous studies have shown that neuroinflammation and oxidative stress are concurrently involved in the pathogenesis of MS. Noticeably, oxidative stress plays a pivotal role in the initiation and progression of MS [90, 117].

Endothelium cells, which act as the interface between the CNS and the periphery, are regulated by ROS in MS patients. High ROS levels induce damage to brain endothelium and dysfunction in the blood-brain barrier (BBB). ROS-mediated increased BBB permeability is followed by the infiltration of leukocytes and transendothelial migration of monocytes into the CNS. ROS perform such an event by changing the phosphorylation state of junctional proteins and cytoskeleton rearrangements, as well as inducing the expression of occludin and ZO1 [23, 118, 119]. Similar to NF-*κ*B, ROS induce the expression of many genes such as TNF-a, inducible nitric oxide synthase (iNOS), cell adhesion molecules (CAMs) such as intracellular adhesion molecules (ICAM-1), vascular cell adhesion molecule 1 (VCAM-1), and platelet endothelial cell adhesion molecule-1 (PECAM-1) in blood-brain barrier-endothelial cells (BBB-ECs) [116, 120]. As well, redox reactions regulate T-cell trafficking into the CNS via activating matrix metalloproteinases. Production of large amounts of ROS by migrating inflammatory cells induces T lymphocytes and monocyte-derived macrophages, which in turn induces neuroinflammation, myelin phagocytosis, oligodendrocyte loss, neuronal and axonal damage, and disease progression in MS [121, 122]. Moreover, microglia, also known as the resident macrophage cells in the CNS, can act in myelin degradation by producing great amounts of superoxide, hydroxyl radicals, hydrogen peroxide, and nitric oxide. NADPH oxidase changes the morphology and proliferation of microglia through generating hydrogen peroxide and is also crucial to the regulation of the expression of several proinflammatory functions of microglia. Therefore, these events trigger the development of neurological pathologies [25, 122, 123]. In addition, the whole-genome microarray analysis has revealed that the p22 subunit of the Nox2 is upregulated in the microglial cells and astrocytes [123]. Oligodendrocytes, a type of glial cells that produce the myelin sheath, are susceptible to oxidative stress due to lower levels of antioxidant enzymes and free radical scavengers and high levels of PUFA and iron. Cellular redox could also inhibit the expression of myelin genes in human primary oligodendrocytes through H_2_O_2_ [124, 125].

The CNS exhibits high oxygen consumption and has a high rate of polyunsaturated fatty acids (PUFAs), which are particularly susceptible to oxidative damage. Myelin sheaths contain 30% protein and 70% lipid, which are vulnerable to lipid peroxidation (LPO) by free radicals, leading to the production of malondialdehyde (MDA), acrolein 4-hydroxy-2-nonenal (HNE), and 4-hydroxy-2-hexenal (4-HHE) [126, 127].

## 6. Antioxidant Systems and Multiple Sclerosis

One of the factors that may regulate the pathogenesis of MS is endogenous antioxidant enzyme systems. Several reports have highlighted the increased expression of antioxidant enzymes, such as SOD1 and SOD2, and catalase in actively demyelinating MS lesions; hence, increased expression of antioxidant enzymes in inflammatory MS lesions is associated with neuroinflammation [117, 128]. The inflammation and cell death in the brain could be regulated by Nrf2. Thus, the activation of the Nrf2 pathway has distinct roles in detoxification of ROS as well as developing MS through regulating levels of certain enzymes [23, 129]. The inflammatory response in the brain and microglial activation increases in Nrf2-deficient mice compared to normal mice. In addition, the severity of the pathology in experimental autoimmune encephalomyelitis (EAE), MS animal model, has been shown to be increased in Nrf2 deficient mice [130, 131]. Treatment with sulforaphane, tBHQ, and Nrf2-inducing agents results in a reduction of neurodegeneration in animal models; therefore, the influences of activation of the Nrf2 pathway in the pathogenesis of MS include (1) decreased leukocyte adhesion by Nrf2 activation and preventing leukocyte infiltration into the CNS; (2) decreased microglial activation by Nrf2 activation and limiting myelin phagocytosis and breakdown via increasing levels of antioxidant enzymes; (3) scavenging ROS by induction of antioxidant enzymes and subsequently reducing the loss of BBB integrity as well as inhibiting oxidative damage to neurons and oligodendrocytes; and (4) induction of oligodendrocyte differentiation by Nrf2 activation via restoring redox balance, thereby reducing demyelination and axonal injury [114, 132, 133].

The SOD, as part of a physiological response to oxidative stress, has pivotal roles in protecting neurons against ROS in MS pathogenesis. Increased gene and protein expression of SOD1 in foamy macrophages and astrocytes and increased SOD2 protein expression in reactive astrocytes have been detected in actively demyelinating MS lesions. The SOD activity has been also presented to be higher in RRMS than controls [128, 134, 135]. The impaired peroxisomal function where catalase is mainly located increases the severity of clinical signs in a rat model for EAE by decreasing both catalase gene expression and activity. Moreover, the upregulation of catalase expression through viral vectors reduces clinical signs in EAE rats. The mRNA expression and activity of catalase have been reported to be increased in MS patients than controls as well [114, 136].

Glutathione peroxidase has been identified with a protective role in neuroinflammation such as MS. The EAE treated with glutathione peroxides restores BBB integrity [137]. Moreover, low concentrations of GSH are important indicators of oxidative stress during the progression of MS [138]. The activity of glutathione peroxidase was reported as an important part of the antioxidant cellular defense system and was found to be increased in the serum of MS patients, along with glutathione reductase activity which is also increased in MS patients. Furthermore, glutathione and *α*-tocopherol concentrations decrease in the demyelinating plaques [116, 138]. Evidence has revealed that the glial cells play pivotal roles in GSH levels. The engagement of the glutathione system in astroglial cells causes diminished antioxidant defense and subsequent neuronal damage [138, 139]. Moreover, the failure of the antioxidant defense occurs due to a genetic defect of glutathione synthesis. The activity of SOD and GPx was reported to be reduced in CD4^+^T cells of MS patients. Moreover, in those with Nrf2 deficiency, differentiation to Th1 and infiltration into the optic nerve is increased [140–142].

Dimethyl fumarate (DMF) has recently been approved as a drug for RRMS, which has anti-inflammatory and neuroprotective effects. DMF and its primary metabolite, monomethyl fumarate (MMF), can improve patient outcomes not only through preventing differentiation of naive CD4 T cells into effector Th1 cells but also by inhibiting cellular infiltration into the CNS, leading to activation of Nrf2-ARE pathway, which is involved in the cellular response to oxidative stress [143–145].

## 7. Conclusion

**Figure 3:**
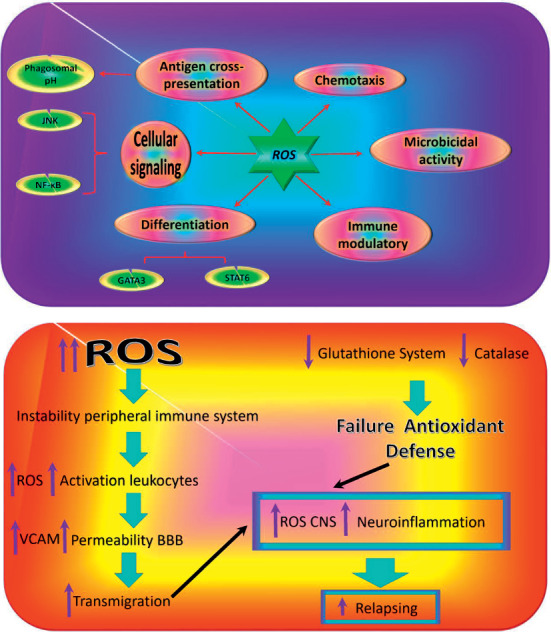
Summary of ROS function in the pathogenesis MS. CNS, central nervous system; VCAM, vascular cell adhesion molecule.

The NADPH oxidase and electron transport chain within the mitochondria contribute to the generation of oxidative species. Contrary to previous studies that showed that ROS can act as a harmful agent on the cellular components such as DNA, protein, and lipid, researchers now have revealed that some of the immune system processes such as proliferation, differentiation, intracellular signaling, chemoattraction, and antigen cross-presentation are regulated by moderate levels of ROS. Though high levels of ROS can be damaging to the immune system, the cellular antioxidant system plays an important role in regulating intracellular levels of ROS. The expression of antioxidant system enzymes is regulated through the Nrf2-Keap1-Cul3 trimeric complex, regulating ROS levels in mammalian cells. The reports have revealed that ROS may be involved in the initiation and progression of autoimmunity diseases such as MS (Figure 3).
